# Regulation of Voluntary Physical Activity Behavior: A Review of Evidence Involving Dopaminergic Pathways in the Brain

**DOI:** 10.3390/brainsci12030333

**Published:** 2022-03-01

**Authors:** Anaissa Ruiz-Tejada, Janet Neisewander, Christos S. Katsanos

**Affiliations:** School of Life Sciences, Arizona State University, Tempe, AZ 85297, USA; aruiztej@asu.edu (A.R.-T.); janet.neisewander@asu.edu (J.N.)

**Keywords:** exercise, motivation, habit, dopamine, neurobiology

## Abstract

Physical activity leads to well-established health benefits. Current efforts to enhance physical activity have targeted mainly socioeconomic factors. However, despite these efforts, only a small number of adults engage in regular physical activity to the point of meeting current recommendations. Evidence collected in rodent models and humans establish a strong central nervous system component that regulates physical activity behavior. In particular, dopaminergic pathways in the central nervous system are among the best-characterized biological mechanisms to date with respect to regulating reward, motivation, and habit formation, which are critical for establishing regular physical activity. Herein, we discuss evidence for a role of brain dopamine in the regulation of voluntary physical activity behavior based on selective breeding and pharmacological studies in rodents, as well as genetic studies in both rodents and humans. While these studies establish a role of dopamine and associated mechanisms in the brain in the regulation of voluntary physical activity behavior, there is clearly need for more research on the underlying biology involved in motivation for physical activity and the formation of a physical activity habit. Such knowledge at the basic science level may ultimately be translated into better strategies to enhance physical activity levels within the society.

## 1. Introduction

Regular physical activity (PA) increases overall quality of life by reducing the risk of metabolic diseases, including cardiovascular disease, type II diabetes, and obesity. The health benefits of PA also extend to neurological diseases including depression and Parkinson’s disease [1]. The World Health Organization has declared physical inactivity as one of the top five leading risk factors for global mortality [2]. The Centers for Disease Control and Prevention advocates for an active lifestyle to prevent diseases originating from day-to-day inactivity, and through the promotion of various strategies at the socioeconomic level (i.e., PA-friendly routes, social support groups, school programs) [3]. The Physical Activity Guidelines for Americans 2018 [4] recommend that adults participate in 150–300 min of moderate-intensity or 75–150 min of vigorous-intensity PA weekly. Approximately 5% of adults engage in at least 30 min of daily PA [5]. Most importantly, the number of individuals meeting PA guidelines has not changed over the years [6]. Current evidence shows that many PA interventions at the socioeconomic level may not be sufficient in improving PA levels [7,8]. Although it is important to focus on an environment that provides opportunities for people to become more physically active, it is imperative to recognize and try to understand the basic biology of the brain that drives implementation of these behaviors. Animal studies over the years have linked certain patterns and brain mechanisms to variations in daily PA [9,10]. Building upon this evidence, recent consensus among experts in the field highlights the importance to understand the brain as a biological determinant of PA [11].

PA can be defined as any locomotion or movement that is the result of skeletal muscle contraction [12]. On the other hand, exercise is defined as a structured, leisure-time recreational and occupational activity [13]. Nonetheless, both PA and exercise result in increased energy expenditure above sedentary levels and mediate improvements in health. For this reason, and for the purpose of this review, PA and exercise will be used interchangeably. The aim of this review is to provide an updated discussion on how dopaminergic systems in the brain are involved in the regulation of voluntary PA behavior. Although there are numerous biological components that work in a coordinated fashion to induce voluntary motor action, we focus specifically on those that control motivation for motor action and current understanding of the brain neurobiology as it relates to reward systems, motivation and habit formation in regulating voluntary PA behavior. We conclude with how this knowledge may be utilized to enhance PA.

## 2. Dopaminergic Pathways in the Brain

Dopamine (DA) is a neurotransmitter with a vast range of functions in the brain. It has been studied considerably in regards to reward and motor control, as well as neurological ailments, such as addiction and Parkinson’s disease. DA in the nucleus accumbens (NAc) regulates motivation and the “wanting” to expend effort to obtain a reward [14]. In addition to its key role in motivation, DA has important roles in cognition [15], as well as motor control and motor pattern formation [16]. To better understand the role of DA in motivation for PA, it is important to briefly discuss the dopaminergic neural circuits involved in PA, motivation, and habit formation.

**Figure 1:**
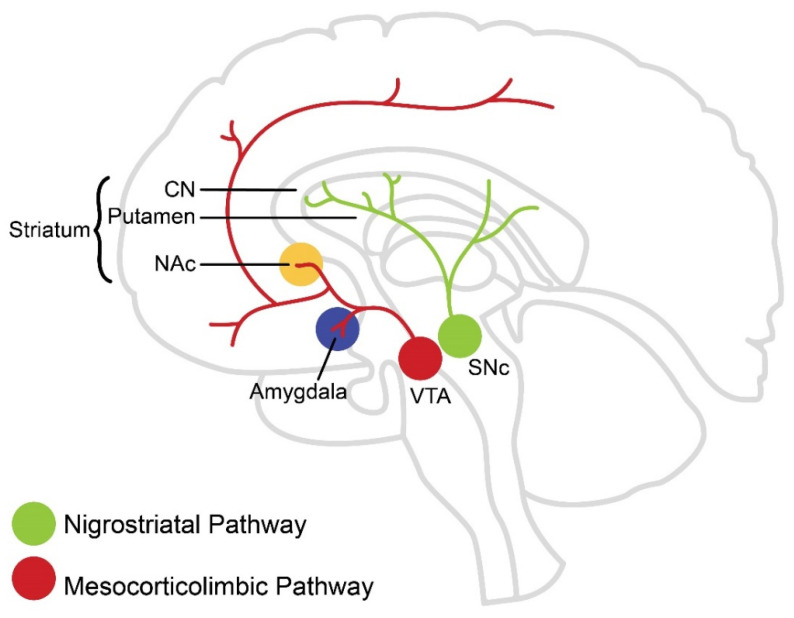
Dopamine Pathways. Dopaminergic neurons originating from the ventral tegmental area (VTA) project to the nucleus accumbens (NAc), amygdala, hippocampus, and prefrontal cortex (PFC) and are referred to as the mesocorticolimbic DA pathway. Dopaminergic neurons originating from the substantia nigra pars compacta (SNc) project primarily to the dorsal striatum and are referred to as the nigrostriatal DA pathway.

Dopamine modulates activity of the basal ganglia, which integrate information flow among cortical, limbic, and motor regions in the brain [16]. They include the caudate nucleus (CN), putamen (Pu), NAc, globus pallidus internus and externus (GPi and GPe, respectively), substantia nigra pars compacta (SNc), and subthalamic nucleus (Figure 1). Interaction and communication between these areas forms a neural network that mediates reward processing [17]. The NAc, CN, and Pu together form the striatum, which is commonly divided into ventral striatum (VS) and dorsal striatum (DS). The VS includes the NAc, which receives input from DA neurons originating from the ventral tegmental area (VTA). These DA neurons comprise the mesolimbic pathway. Other VTA dopamine neurons project to the amygdala, hippocampus, and prefrontal cortex, and collectively VTA projections are referred to as the mesocorticolimbic pathway. The DS receives dense input from DA neurons originating primarily from the SNc [18]. These DA neurons make up the nigrostriatal pathway. Although both the mesocorticolimbic and nigrostriatal pathways (Figure 1) are associated with motor control as well as reward and habit learning [19,20], a report by Boekhoudt and colleagues [21] shows that chemogenetic activation of dopamine neurons (i.e., tyrosine hydroxylase neurons) in VTA, but not SNc, induces a hyperactive phenotype in rats. This suggests that neurons originating from the VTA are likely mostly associated with voluntary PA behavior.

DA produces its effects by binding to G-protein-coupled receptors (i.e., D1-like and D2-like receptors). D1-like receptors include D1 and D5 receptors, and D2-like receptors include D2, D3, and D4 receptors. The D1- and D2-like receptor families have distinct anatomical distributions and intracellular signaling effects, and have been implicated in regulating motivation and motor control of voluntary PA. Action of DA in the synapse is terminated primarily through reuptake of DA by dopamine transporters (DAT), although some DA is metabolized in the synapse.

## 3. Evidence for Regulation of Motivated Physical Activity Behavior through Dopaminergic Pathways

### 3.1. Selective Breeding and Pharmacological Studies in Rodents

**Why rodents choose to run in wheels has been a topic of great interest to the field of basic exercise neuroscience for a long time.** Various theories have been proposed to affect physical activity in rodents and related to both internal and external factors (e.g., foraging, stereotypy, reinforcement, environmental complexity, light), and which have been previously reviewed in detail [22,23]. Despite the complexity of the factors that drive PA in rodents, studies employing rodent models have provided over the years insights into possible factors regulating PA behavior. A model frequently employed to enhance our knowledge into the biology that drives increased PA behavior involves rodents selectively bred for high levels of PA. Examining wheel running and spontaneous PA in such rodents can provide insights into the neurobiology underlying spontaneous PA [23]. Researchers have utilized high- and low-PA rodent models to compare effects of pharmacological agents affecting dopaminergic pathways on PA running behavior (Table 1).

Interestingly, systemic administration of either a selective D1-like receptor agonist or a selective D1-like receptor antagonist decreases overall wheel running (distance and/or duration) in mice characterized by high levels of PA, with no effects on low PA mice [24,25]. Similarly, Roberts et al. [26] report reduced voluntary wheel running in high-voluntary running rats administered either a D1-like receptor agonist or antagonist directly into the NAc. This suggests that D1-like receptor modulation in the NAc plays a role in running behavior in high-PA rodents. A limitation, however, is that it is not clear whether the high and low running levels of experimental, inbred rodents can translate to individual differences in PA in humans. However, in the absence of more pertinent evidence, these lines of evidence support a role of distinct D1-like receptor function on voluntary PA that may be dependent on baseline and/or genetic predisposition for PA behavior.

An explanation for the decrease in voluntary running by both D1 agonist and antagonist drugs is that either enhancing or preventing transduction at the D1-like dopamine receptors decreases motivation to run. Similarly, Alleweireldt and colleages found that administration of D1-like receptor agonists and antagonists decreases cocaine-seeking behavior [27,28]. Because the latter behavior reflects motivation for cocaine, these drugs may have analogous effects on motivation for PA. Although the similar effects on PA behavior in response to D1-like receptor agonist and antagonist are not easy to reconcile, it is possible that these pharmacological agents interact with different populations of receptors including nonspecific sites [27]. Another explanation is that D1 agonists induce grooming behavior, which may interfere with voluntary running [27].

Although forced running differs from voluntary running in terms of brain adaptations and psychological state [29], systemic administration of D1-like and D2-like receptor antagonists either separately or in combination decreases forced running wheel behavior. However, when administered directly into the striatum of rodents, only the D1-like receptor antagonist decreases the duration of forced running wheel performance [30]. These findings implicate a role of extra-striatal D2 receptors and intra-striatal D1 receptors [30]. Moreover, these findings highlight that in order to understand the role of DA in regulating motivated PA behavior, research needs to employ receptor location-specific experimental approaches within the mesocorticolimbic pathway. Another important factor in such studies is dose, particularly when investigating the role of D2-like receptors given that low doses of D2-like receptor drugs may preferentially bind to D2-like autoreceptors including the high-affinity dopamine D3 receptors, whereas higher doses are needed to bind to post-synaptic D2 receptors.

Zhu et al. [31] employed Designer Receptors Exclusively Activated by Designer Drugs (DREADD)-based experimental tools [32] to understand the role of D1- and D2-receptor-expressing neurons in the NAc in modulating PA. DREADDs are modified muscarinic G-protein-coupled receptors that include Gq or Gi proteins. The ligand clozapine-N-oxide is the designer drug that activates Gq or Gi signaling, resulting in neuronal activation or silencing, respectively [33]. Injection of adeno-associated virus expressing Gq- or Gi-coupled Cre-inducible DREADD into the NAc of D1-Cre or D2-Cre transgenic mice allows the activation/deactivation of neurons expressing D1 and D2 receptors, respectively. Using this experimental approach, Zhu et al. found opposite effects in these two lines of transgenic mice. The activation of D1-receptor-expressing neurons increases PA (i.e., voluntary wheel running/locomotion), while activation of D2-receptor-expressing neurons reduces PA [31]. Moreover, inhibition of D2-receptor-expressing neurons increases PA [31], suggesting that the degree of D2 receptor activity is inversely related to PA. Future research is needed to determine the specific subpopulations of D1- and D2-expressing neurons that mediate the observed effects given that there is heterogeneity among both types of neurons.

The increase in PA by activation of D1-receptor-expressing neurons appears to contradict the previous findings that D1 receptor agonist decreases PA [26]. These opposite effects are not easy to reconcile, but they may be due to a nonspecific effect of either DREADDs or the D1 receptor agonist. It is also possible that the use of rodent inbred for low and high voluntary running used in the agonist study may not be the most appropriate model when compared to wild-type rodents to unravel the specific role of D1 receptors on the regulation of voluntary PA. Other advantages of the Zhu et al. [31] study stem from not only targeting the NAc but also dissecting specific roles of D1- versus D2-expressing neurons in modulating PA behavior. Thus, D2 receptors in NAc may have a primary role, greater than previously thought, in modulating motivated PA behavior.

### 3.2. Genetic Studies in Rodents and Humans

Genetic influences play key role in regulating PA behavior [34]. The relevant literature implicating heritability of PA in humans and rodents has already been reviewed [35]. For the purpose of this review, we are discussing evidence of genes related specifically to dopaminergic pathways and motivated PA behavior. This evidence relates to gene expression in high PA and low PA rodents, as well as to gene and single nucleotide polymorphisms (SNP) in humans. These genes control DA signaling, transmission, or availability, as well as the expression of signaling molecules that indirectly regulate dopaminergic pathways involved in motivated PA behavior.

Evidence from rodent studies shows that the DA receptor D1 (Drd1) gene has an important role in regulating voluntary PA behavior [35], and it is differentially expressed in the NAc of high PA versus low PA mice. Specifically, Drd1 is lower in the strain characterized by high levels of PA and higher in that characterized by low levels of PA [36,37]. Relevant to this evidence, the transcription factor AP-2α (Tcfap2a) appears to have a key role in regulating voluntary PA because it controls the promoter activity of Drd1 [37]. However, some studies have not found differences in expression of DA D1 receptors in the NAc of rodents characterized by high versus low PA [38]. It is likely that differences in PA may not relate necessarily to the expression of the Drd1 gene, and that D2-expressing neurons in the NAc also regulate PA.

In humans, individuals with rs6275 SNP in the DRD2 gene are characterized by increased time performing moderate-to-vigorous PA in response to a PA intervention program [39]. A separate genotype associated with DRD2 is the rs1800497 polymorphism, known as Taq1A allele, which is associated with low D2 receptor density [40], and is also linked to increased likelihood of engaging in PA [41]. Moreover, the association between Taq1A and D2 receptors is regulated by ankyrin repeat and the kinase domain containing 1 (ANKK1) gene [42], which is also linked to dopaminergic deficiency in the striatum [43]. Thus, the findings on polymorphisms linked to the DRD2 gene, together with the rodent studies discussed above [31], strongly implicate that D2 dopamine receptor mechanisms play a role in voluntary PA.

**Figure 2:**
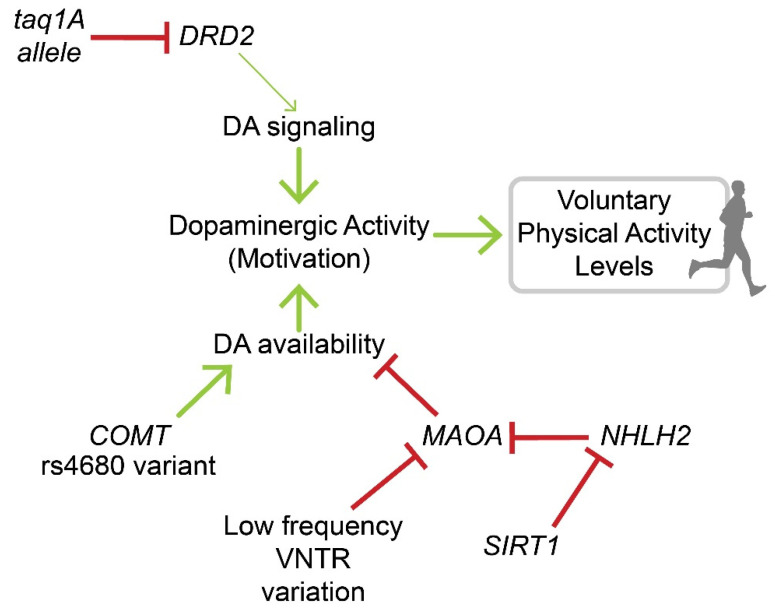
Genetic regulation of voluntary physical activity through dopamine pathways in the brain. A proposed model depicting genetic evidence-based potential biological mechanisms involved in the regulation of motivation for physical activity via dopamine-associated pathways (see text for explanation of genes shown; arrow head indicates procession, with the size of the arrow representing degree of procession; T head indicates inhibition/reduction).

In terms of genes that affect DA availability, catechol-O-methyltransferase (COMT) is of particular importance because of its enzymatic role in DA metabolism [44]. A polymorphism in the COMT gene (rs4680) associated with slower clearance of dopamine, and thus higher levels of dopamine at the synapse, is linked to increased time performing moderate-to-vigorous PA following a PA intervention program [39]. The enzyme monoamine oxidase A (MAOA) also affects DA availability and behavior in both humans and rodents through metabolism of monoamines, including DA [45]. MAOA contains a polymorphism of a variable number of tandem repeats (VNTR) that affects transcription rates. A low frequency (3 repeats) variant results in decreased MAOA expression, while a high frequency (3.5–4 repeats) variant results in increased MAOA expression [46]. Good et al. [47] reported an association between the number of repeats and levels of PA, with low frequency variants linked to higher PA levels, while high frequency variants were associated with lower PA. On the other hand, Goleva-Fjellet et al. [48] did not find a significant association between the MAOA VNTR polymorphism and PA levels, which may be explained by a reported lack of association between MAOA genotype and brain MAOA activity [49]. MAOA is also inhibited by the nescient helix-loop-helix 2 (NHLH2) protein, and expression of NHLH2 is inhibited by sirtuin 1 (SIRT1) [50]. These effects of NHLH2 on MAOA activation suggest that NHLH2 may also have a role in DA-mediated regulation of PA. However, this research remains to be done to determine whether this pathway impacts PA. A summary of the genetic evidence discussed above and a proposed model showing potential mechanisms involved in motivation for PA via dopamine-associated pathways is shown in Figure 2.

## 4. Evidence for Regulation of Physical Activity Habit Formation through Dopaminergic Pathways

Occasional motivation for and performance of PA is not enough to obtain the benefits resulting from PA; the benefits require this behavior to become habitual resulting in regular/repeated PA. Although motivation controls goal-directed behavior that is dependent on a reward [51], habit is formed as a result of repetition and may eventually become insensitive to reward (i.e., behavior continues despite reward devaluation) [52]. As opposed to motivation that may occur occasionally, habit is a sequential and repetitive behavior triggered by specific cues without conscious cost-benefit analysis [53]. Regarding a PA habit, research to date has focused on the psychology of PA habit formation. This habit formation for PA starts with the need and/or desire to exercise, followed by an assessment of individual environment factors (including skills and social pressure), and then outcome evaluation. Satisfactory outcome reinforces repetition leading eventually to formation of habit for PA [54]. Relevant studies show that the strength of a habit formation is correlated with repetition and positive reinforcement [54], as well as intrinsic levels of motivation [55].

The psychology of PA habit formation suggests the existence of underlying biology supporting the formation of a PA habit. However, there is a lack of research about the brain biological mechanisms involved in PA habit formation. Outside the PA research field, there is ample evidence implicating dopaminergic pathways in the regulation of habit formation, as well as the transition between motivated and habit-formed behavior [52,56,57]. The neurobiology of motivation, which was discussed in the previous section, is regulated mainly in the mesolimbic DA pathway. However, experimental evidence shows that lesions in the SNc inhibit the formation of habits [58], suggesting that habit formation is regulated mainly by the nigrostriatal DA pathway (Figure 1). In terms of anatomy, motivated behavior takes place at the dorsomedial striatum (DMS) [59], but habit formation takes place in the dorsolateral striatum (DLS) [56,60]. In primates, the DLS is known to receive input from sensory-motor areas, while the central striatum, receives input from associative cortical areas [17].

Amphetamine, which elevates brain extracellular DA concentrations [61], increases locomotion in rodents. This effect sensitizes with repeated amphetamine experience and increases habit formation [62], supporting a key role of DA. The primary mechanism by which amphetamine increases extracellular dopamine is by reversing DAT, resulting in release of DA rather than reuptake of striatal DA [63]. Mice administered a D2 antagonist (i.e., eticlopride) had enhanced habit formation [64]. Moreover, there is evidence using instrumental conditioning in mice that habit formation involves an inhibition of movement by D2 activation in the DLS and that post-synaptic plasticity at D2-expressing neurons is key in mediating habit formation in the DLS [65]. Overall, this evidence highlights the role of dopaminergic pathways in the process of habit formation with possible implications for PA habit formation. Consequently, there is a pressing need for research to better understand the neurobiology of habit formation as it relates to PA.

## 5. Biological Signals Supplementing Dopaminergic Regulation of Physical Activity Behavior

As discussed above, the dopaminergic mesocorticolimbic pathways play a role in the regulation of motivation for PA. In addition to DA, other signaling molecules can regulate motivation for PA. This section will focus on such biological signals interacting with the dopaminergic system, to emphasize their link to dopaminergic-mediated PA, as well as stimulate relevant research in the field.

### 5.1. Serotonin

Serotonin (5-HT) is a neurotransmitter that modulates DA availability through a variety of 5-HT receptor subtypes located on dopaminergic neurons or by interacting with GABAergic or glutamatergic neurons [66]. Blocking the 5-HT2C receptor in rodents increases DA at the NAc [67]. Mice selectively bred to be high runners have lower concentrations of 5-HT in the dorsal striatum as compared to controls [68]. High dose of a combination of 5-HT1A agonist and a 5-HT1A/1B partial agonist reduces voluntary wheel running in rats characterized by high levels of wheel running [69]. Thus, 5-HT and its receptors modulate DA availability in areas of the brain that regulate motivated behavior, including PA behavior. In this regard, fatigue, lethargy, and tiredness are associated with a higher ratio of 5-HT to DA in the striatum [70], with possible implications for motivation for PA via the dopaminergic pathway.

### 5.2. Leptin, Ghrelin, and Insulin

The hormones leptin, ghrelin, and insulin have been implicated mostly in feeding behavior. Leptin modulates mesolimbic DA through leptin receptors (LepR) found on dopaminergic neurons in the VTA [71], and where signaling through these receptors decreases dopamine neuronal activity [72]. Although the expectation would be that increased leptin signaling decreases PA, direct administration of leptin to the VTA in rodents does not lower locomotor activity [72]. However, knockdown of LepR in the VTA in rodents increases locomotor activity [72], still implicating leptin signaling in the VTA as an important regulator of PA. A model by which impaired dopaminergic activity in obesity results in reduced PA, as well as the role of central leptin resistance in obesity in this response, has been recently proposed [73]. The overall evidence to date clearly highlights an important role of leptin in regulating voluntary PA through dopaminergic pathways, but more research is needed to clarify its exact role in regulating voluntary PA in humans.

As opposed to leptin, ghrelin signals energy depletion (i.e., hunger) by acting on its receptor, the growth hormone secretagogue receptor (GHSR), which is expressed in dopaminergic neurons of the SN and VTA [74]. Thus, ghrelin activates the brain reward pathways to increase food intake, leading to increased locomotion via a “food-anticipatory activity” mechanism [75]. Regarding its effects on PA, ghrelin increases motivation and hyperactivity [76].

Insulin is linked to reduced motivation for food after eating (i.e., satiety signal) [77]. It modulates the rewarding aspects of food via the DA mesolimbic pathway [78]. Although specific evidence is currently lacking, it is reasonable to postulate a role of insulin in regulating motivated PA behavior because insulin and its receptors modulate DA release in the VTA and NAc. Insulin effects on overall dopaminergic activity have previously been reviewed by Sallam and Borgland [79].

### 5.3. Estrogen

It has been known for some time that estrogen regulates DA release and transmission within the basal ganglia [80]. Current evidence corroborates the role of estrogen in regulating voluntary PA. Available evidence suggests that overall activation of the estrogen receptor (ER)α-pathway by estrogen increases spontaneous PA, which has been previously reviewed in detail [81]. Current evidence, however, from Krentzel et al. [82] shows that estradiol acting through α and ß ERs can both decrease and increase wheel running in rodents via rapid (i.e., acting as neuromodulator) and non-rapid (i.e., acting as hormone) signaling, respectively. More research is needed to unravel the exact roles of estrogen on modulating PA via dopaminergic pathways.

### 5.4. Endocannabinoids and Orexins

Endocannabinoid and orexin signals interact in the brain to regulate various behavioral processes including feeding behavior and energy balance [83,84]. Endocannabinoids achieve this by modulating brain DA [85]. Sallam and Borgland [79] describe the role of endocannabinoids in mediating plasticity at the mesolimbic pathways though its interactions with DA and GABAergic neurons. Endocannabinoids are active in the striatum [86], act mostly through CB1 receptors [87], and are associated with motivation for PA in rodents [88]. Mechanisms that drive habit formation and reward via endocannabinoid signaling have been reviewed by Hilário and Costa [89] and Parsons and Hurd [90], respectively. In addition, Sallam and Borgland [79] have described the role of endocannabinoids in mediating plasticity at the mesolimbic pathways through its interactions with DA and GABAergic neurons. Supporting a role of endocannabinoids in regulating PA, CB1-receptor-deficient mice have impaired PA responses [91], and mice that are bred for high running capacity decrease their running in response to CB1 receptor antagonism [92]. Besides their role in motivation, signaling through endocannabinoids is implicated in habit formation as shown by decreased habit formation in mice with a CB1 polymorphism or mutation [86].

Orexins are neuropeptides localized mostly within the hypothalamus [93]. Orexin neurons project into the VTA and activate mesolimbic DA pathways via mechanisms that have been previously described [94]. Orexin signaling is linked to motivated behaviors, including feeding and spontaneous PA [94,95].

## 6. Applicability of the Findings on the Role of Dopamine in Regulating Physical Activity Behavior

Despite being limited, available knowledge on the role of DA and dopaminergic pathways in modulating PA has important implications for establishing healthy lifestyles. At the basic science level, understanding the role of DA in regulating PA is important to stimulate translational research in the field of PA behavior. A high monoamine diet or consumption of cocoa extract increases the plasma levels of the DA metabolite homovanillic acid (HVA) [96,97]. However, it has yet to be determined whether dietary manipulations impact the activity of dopaminergic pathways in the brain in a way that regulates voluntary PA behavior. Nevertheless, diet has profound effects on PA behavior, and it has been shown that a diet high in fat/sugar reduces voluntary wheel running in rodents [98]. Moreover, the metabolic state of obesity, commonly associated with increased caloric/fat intake, correlates with low levels of PA [99]. Kravitz et al. [99] have reviewed the interactions between obesity and PA and suggest that high fat diets induce deficiencies in striatal DA signaling, leading to lower PA levels in humans. Studies in rodents show that consumption of saturated, but not monounsaturated fat, reduces mesolimbic DA signaling and blunts the reward response [100]. In humans, replacing dietary saturated fat with monounsaturated fat leads to increased levels of daily PA [101]. Collectively, these lines of evidence describe effects of dietary intake on modifying dopaminergic pathways in the brain with implications for PA behavior.

Caffeine has long been the most used psychostimulant [102]. The effects of caffeine result from blocking the adenosine A1 and A2A receptors, and antagonizing these receptors enhances DA signaling in the brain [103]. Antagonism of the adenosine A1 receptor enhances the effects of DA D1 receptor agonists in brain [102,104], underscoring the role of caffeine in enhancing dopaminergic pathways in the central nervous system. In mice, Claghorn et al. [105] showed that caffeine increases voluntary PA. In humans, caffeine consumption is linked to increased duration of performing exercise, along with some effect of caffeine on increasing exercise enjoyment [106]. More research with respect to the underlying biology supporting these observations in humans will provide evidence about the exact role of caffeine consumption in regulating dopaminergic pathways in the brain that enhance motivation for PA.

PA is a natural reward that produces adaptations in both rodents and humans consistent with an increase in evoked DA release [107,108]. Therefore, it is reasonable to expect that initiating some form of PA induces biological adaptations within brain dopaminergic pathways that establish motivation for PA as well as formation of a PA habit. In support of this notion, current evidence shows that repeated participation in PA increases enjoyment associated with such activity [106]. Future research may investigate types and aspects of PA (i.e., intensity, duration) that are employed in a way that upregulates dopaminergic pathways linked to increased spontaneous PA behavior.

Transcranial direct current stimulation (tDCS) involves non-invasive neuromodulation which has been used successfully in research to regulate food intake behavior [109]. In regards to PA, tDCS reduces perceived effort and enhances performance [110]. Recent research by Fonteneau et al. [111] shows that tDCS increases DA levels in the striatum of humans, opening a range of possibilities for neuromodulation to potentially regulate brain dopaminergic pathways related to human behavior, including PA. Future research may determine the applicability of this technique and the conditions under which neuromodulation increases brain DA levels and enhances spontaneous PA behavior.

## 7. Conclusions

Attempts to enhance PA behavior in our society are incomplete without an in-depth understanding of the underlying biology that determines motivation for PA and PA habit formation. As discussed in this review, current evidence establishes an important role of dopaminergic pathways in the brain in determining PA behavior. A major limitation to our understanding of the mechanisms that regulate voluntary PA behavior in humans is that most research to date has been performed in rodents. Although rodents offer several experimental advantages over humans (i.e., precise experimental manipulations), results from these studies are always subject to interpretation based on the rodent breeding protocols used as well as the specific conditions of each experiment, such as early exposure to a running wheel. Expanding our basic science knowledge regarding the brain dopaminergic pathways regulating PA behavior in humans is key to enhancing PA in our society.

## Figures and Tables

**Table 1:** Experimentally employed pharmacological agents targeting dopaminergic pathways and physical activity.

Reference	Rodent Model	Pharmacological Agent (Mode of Action)	Route of Administration	High Activity	Low Activity/Control Strain
				Strain	
Rhodes et al., 2001	Mouse	Cocaine (non-selective DAT antagonist)	Systemic	↓ WR (speed)	no overall effect
		GBR 12909 (DAT antagonist)		↓ WR (speed)	no overall effect
		Fluoxetine (Prozac) (non-selective DAT antagonist)		↓ WR (speed and duration)	↓ WR (speed and duration)
Rhodes and Garland, 2003	Mouse	Methylphenidate (Ritalin) (non-selective DAT antagonist)	Systemic	↓ WR (distance)	↑ WR (distance)
		Apomorphine (non-selective DA agonist)		↓ WR (distance)	↓ WR (distance)
		SCH 23390 (D1-like DA antagonist)		↓ WR	↓ WR
		Raclopride (D2-like DA antagonist)		↓ WR	↓ WR
Knab et al., 2012	Mouse	SKF 81297 (D1-like DA agonist)	Systemic	↓ WR	no overall effect
		SCH 23390 (D1-like DA antagonist)		↓ duration	no overall effect
		GBR 12909 (DAT antagonist)		no overall effect	↑ WR
		AMPT (tyrosine hydroxilase inhibitor)		↓ duration	no overall effect
Roberts et al., 2012	Rat	SKF 81297 (D1-like DA agonist)	Bilateral injection to NAc	↓ WR (distance)	no overall effect
		SCH 23390 (D1-like DA antagonist)		↓ WR (distance)	no overall effect
Toval et al., 2021	Rat	Raclopride (D2-like DA antagonist) SCH 23390 (D1-like DA antagonist) Raclopride (D2-like DA antagonist) SCH 23390 (D1-like DA antagonist)		**Effect on Forced Running**	**Effect on Open Field Test**
			Systemic	↓ duration	↓ locomotor behavior
				↓ duration	↓ locomotor behavior
			Bilateral injection to DS	no overall effect	no overall effect
				↓ duration	no overall effect

DAT, dopamine transporter; WR, wheel running; NAc, nucleus accumbens; DS, dorsal striatum.
